# Paraganglioma in a Young Adult Female Patient: A Case Report

**DOI:** 10.7759/cureus.36963

**Published:** 2023-03-31

**Authors:** Christopher M Stevens, Kevin Malone, Reena D Wadhwa, Nathan A Rinehouse, Amro Saad Aldine, Guillermo Sangster

**Affiliations:** 1 Interventional Radiology, Louisiana State University Health Sciences Center, Shreveport, USA; 2 Biomedical Engineering, Louisiana State University Health Sciences Center, Shreveport, USA; 3 Radiology, Louisiana State University Health Sciences Center, Shreveport, USA; 4 Pathology, Louisiana State University Health Sciences Center, Shreveport, USA; 5 Interventional Radiology, University of Texas Southwestern Medical Center, Shreveport, USA

**Keywords:** malignancy surgery, catecholamine hypersecretion, tumor imaging, neuroendocrine tumors, paraganglioma

## Abstract

Paragangliomas are catecholamine-secreting neuroendocrine tumors that originate from the chromaffin cells of the sympathetic ganglia. Roughly 10% of paragangliomas are malignant, resulting in a rare occurrence of 90-95 cases per 400 million people. Herein, we report a case of a 29-year-old female patient who presented with nausea, vomiting, and bloating and was found to have a large left retroperitoneal tumor upon imaging. The tumor was successfully removed, and subsequent histological analysis was compatible with the presence of a paraganglioma. This case serves as a reminder that despite its rarity, paragangliomas should never be dismissed as a differential diagnosis if correlating symptoms and diagnostic findings are consistent with that of paraganglioma etiology.

## Introduction

Paragangliomas (PGs) and pheochromocytomas (PCs) are catecholamine-secreting neuroendocrine tumors that originate from the chromaffin cells of the sympathetic ganglia [1]. PGs share an almost identical profile with PCs, with the anatomical location of the tumors, PGs being extra-adrenal tumors and PCs growing inside the adrenal gland, being the only differentiation between the two [2]. PG symptomatology can vary greatly but commonly includes headaches, diaphoresis, and tachycardia. Panic attacks, palpitations, weight loss, hyperglycemia, dyspnea [1], and exercise-induced nausea and vomiting in young adults [3] have been reported as well. Surgical excision of the tumor is the gold standard for treatment.

PGs and PCs are mostly benign, with only 10% being malignant [1]. Herein, we report a case of a 29-year-old female patient who presented with nausea, vomiting, and bloating and was found to have a left extra-adrenal mass upon imaging that appeared to be malignant. The tumor was successfully removed, and subsequent histological analysis was compatible with the presence of a PG. This case serves as a reminder that despite their rarity, PGs should never be dismissed as a differential diagnosis if correlating symptoms and diagnostic findings are consistent with that of PG etiology.

## Case presentation

**Figure 1:**
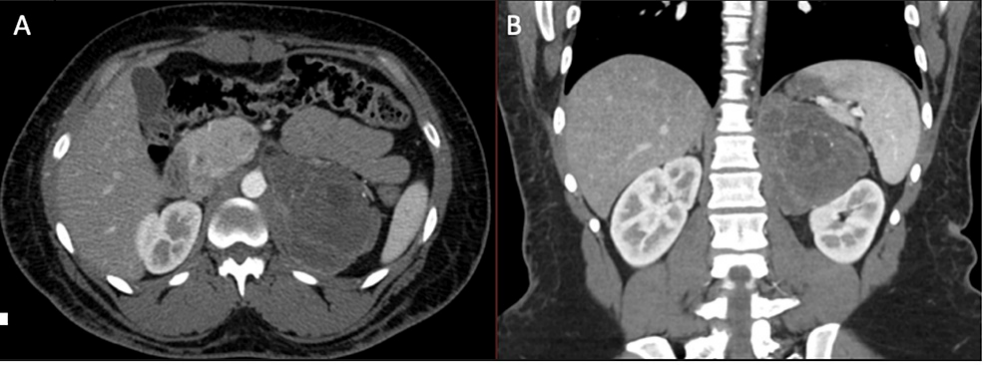
Axial (A) and coronal (B) contrast-enhanced CT images show a large left retroperitoneal mass with multiloculated cystic and necrotic components, thin enhancing walls/septa, and isolated punctate calcifications. There is a mass effect on the upper pole of the left kidney.

A 29-year-old, African American female, with no significant past medical history or current medication use, presented to the emergency room with complaints of nausea, vomiting, and bloating. A computed tomography (CT) of the abdomen with contrast was ordered, which showed the presence of a left retroperitoneal mass approximately 6.8 x 9.5 x 1.4 cm in size with necrotic components (Figure 1). The leading diagnosis at this point was an adrenal cystic lymphangioma. The patient was referred to urology where a functional workup was negative for significant findings. Results were as follows: aldosterone 3.8 ng/dL (Lab ref. 23.2 ng/dL), renin 1.4 ng/mL/h (Lab ref. 0.6-4.3 ng/mL/h), cortisol 17 mcg/24hr urine (Lab ref. 3.5-45 mcg/24hr), metanephrines 65 mcg/24hr urine (Lab ref. 30-180 mcg/24hr normotensive; <400 mcg/24hr hypertensive), normetanephrine 203 mcg/24hr urine (Lab ref. 103-390 mcg/24hr normotensive; <900 mcg/24hr hypertensive), total metanephrine 268 mcg/24hr urine (Lab ref. 145-510 mcg/24hr normotensive; <1300 mcg/24hr hypertensive), with a urine total volume of 750 ml in 24 hours. The complete blood count and comprehensive metabolic panel were normal. Re-evaluation for mass and lung metastasis rule-out was repeated with CT chest without contrast and CT abdomen with and without contrast, but no masses were found. The patient was then scheduled for an open left adrenalectomy overlying the ninth rib (ninth rib resection and diaphragm repair) with the placement of a chest tube.

**Figure 2:**
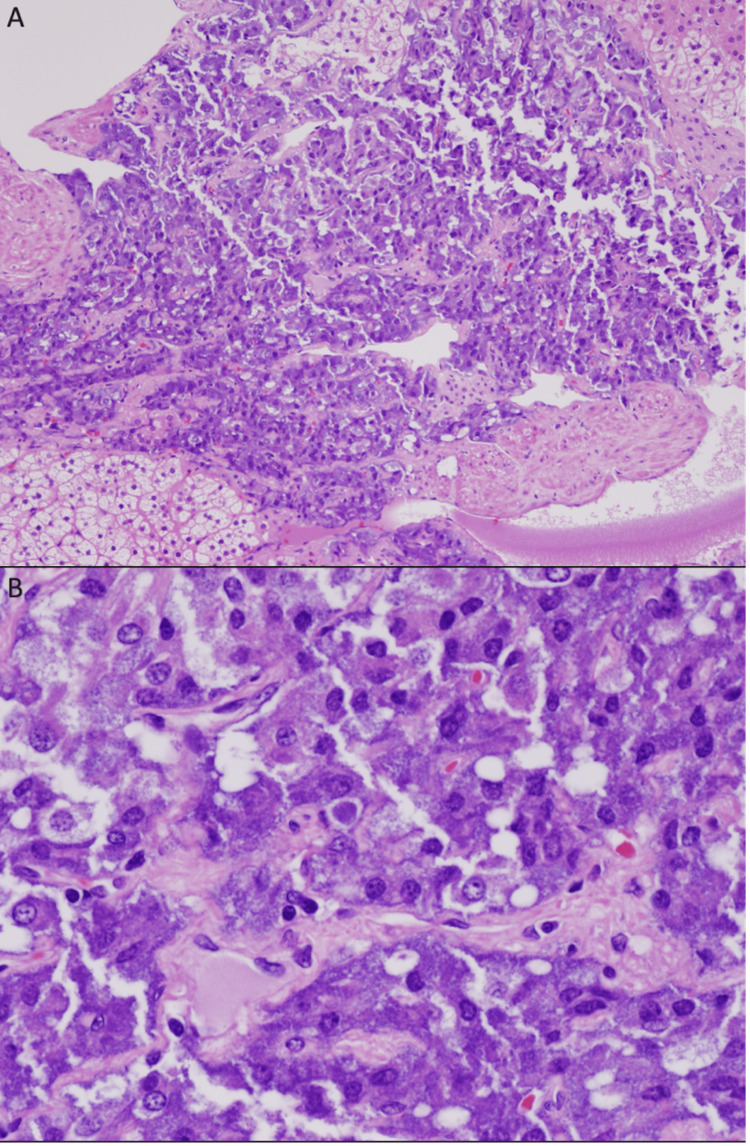
(A) Intermediate magnification (100x) H&E photomicrograph demonstrating large epithelioid cells arranged in a somewhat nested fashion. These cells have a palely eosinophilic granular cytoplasm and notably atypical vesicular nuclei; (B) High magnification (400x) H&E photomicrograph demonstrating a cytologically malignant neoplasm consisting of quite large epithelioid cells arranged in a somewhat nested fashion. These cells have a palely eosinophilic granular cytoplasm and notably atypical vesicular nuclei.

The patient underwent a successful operation without complications. Throughout the surgery and for three days postoperative, the patient maintained normotensive status with systolic pressures ranging primarily from 120s-140s systolic and 50s-60s diastolic. The patient did not receive alpha or beta blockage, which had a good outcome in this particular case. The excised specimen was sent for pathologic examination, which microscopically showed a portion of the sympathetic chain with multifocally scattered ganglion cells and a neoplasm consisting of large epithelioid cells arranged in a nested fashion (Figure 2). The mass appeared to abut the adrenal gland but not invade it. NF, SOX-10, and SDHB staining were all positive. MYCN gene (N-MYC) amplification was negative for the tested specimen. Staining for PHOX2B was negative, ruling out ganglioneuroblastoma. Staining for CD34 and caldesmon was also negative. No mast cells were seen with the c-KIT stain. The present tumor was identified as favoring malignant due to its imaging profile demonstrating local invasion and necrotic components. No postoperative complications occurred, and the patient was discharged five days after the procedure with plans to re-image six months later.

## Discussion

PGs are a rare type of hypervascular neuroendocrine tumor capable of synthesizing and releasing catecholamines (dopamine, norepinephrine, and epinephrine) [4]. Approximately 500-1,000 cases of PGs are diagnosed per year in the United States, thus highlighting its rarity [5]; however, the incidence rates of PGs have increased over the last 20 years due to an intensified use of biochemical tests and imaging studies [6]. Most PGs are benign, with malignant PGs only having 90-95 occurrences per 400 million people [5]. Herein, we present a case of a malignant PG in a young adult female patient. Imaging and histological analysis were beneficial in diagnosing the tumor type, and surgical removal of the mass was successful in treatment. This manuscript serves as a reminder to never disregard a medical diagnosis due to its rarity while also adding another presentation, diagnosis, and treatment of a PG to the existing body of literature.

PGs have a strong genetic susceptibility and approximately 35% are hereditary [7]. Familial PGs are often bilateral and occur at a younger age than sporadic PGs. Approximately 25% of all carriers will show expression of the tumor before the age of 25 [8]. Males and females are affected at equal rates by the familial form due to an autosomal dominant inheritance type [8]. Most mutations occur in the DNA of genes encoding for the protein succinate dehydrogenase (SDH), an enzyme found in complex II of the respiratory chain in the mitochondria [8,9]. Tumor syndromes related to PGs include multiple endocrine neoplasia type 2 (MEN 2), von Hippel-Lindau (VHL), and neurofibromatosis type 1 (NF-1) [8]. Due to the strong hereditary association, taking a family history is vital in these patients. In our case, the patient had an unremarkable family history and a unilateral PG, thus meaning the PG was likely sporadic in nature.

PGs can be difficult to diagnose due to the diverse presentation they can exhibit [5]. Pathogenesis is due to the release of catecholamines, predominantly norepinephrine, and can result in various manifestations, including gastrointestinal (chronic constipation), cardiovascular (chest pain and tachycardia), neurologic (hoarseness, dilated pupils, cranial nerve paralysis), endocrine (polyuria and polydipsia), psychiatric (panic mode and fidgetiness), musculoskeletal (fatigue), and urological (micturition syncope) [5]; however, the most common presentation of PGs is a triad of headache, palpitations, and profuse sweating [2].

Along with symptomology, biochemical testing and histological analysis help in diagnosing PGs. Biochemical diagnosis involves the measurement of urinary and plasma catecholamines, urinary fractionated metanephrines, plasma-free metanephrines, and urinary vanillylmandelic acid [10]. Evaluating the concentration of metanephrines, the metabolic intermediate of catecholamines, is considered a superior method for diagnosing PGs and PCs compared to measuring catecholamines due to the longer half-life metanephrines possess [10-12]. In the described patient, functional testing included plasma renin, aldosterone, 24-hour urinary cortisol, and metanephrines. It is important to note though that medications, including tricyclic antidepressants, antipsychotic agents, serotonin-reuptake or norepinephrine-reuptake inhibitors, and levodopa, can increase catecholamine levels, causing a false positive test result [2]. To prevent this, these medications should be tapered and discontinued at least two weeks before the biochemical assessment is performed [2]. Histologically, PGs produce a Zellballen pattern due to the appearance of polygonal chief cells in small clusters that are nested within surrounding sustentacular cells, glial-like cells associated with chromaffin cells that are S-100 positive [13-16]. No histological criteria exist for distinguishing between benign and malignant PGs. PGs are said to be malignant only when cells displaying neoplastic characteristics are found in areas where paraganglionic tissue is usually absent [17]. In the present report, the tumor was identified as malignant due to the presence of local invasion and necrotic components.

The use of imaging is also important in helping locate and characterize PGs as benign or metastatic and is usually performed after biochemical tests have confirmed the presence of excess catecholamines [2,18]. The combined use of CT, magnet resonance imaging (MRI), and nuclear medicine exhibits a high sensitivity rate, almost 100%, for diagnosing catecholamine-producing tumors [19-21]. On CT, PG and PC masses commonly appear as a nonenhanced density greater than 10 Hounsefield units with cystic changes, internal calcifications, and necrosis [22,23]. MRI is recommended over CT in the pediatric population due to radiation exposure to CT. The sensitivity of CT has also been reported to be low and inferior to MRI for the detection of PGs [24]. The cystic components and internal hemorrhage regularly seen in these tumors result in a heterogeneous MRI appearance [22]. On MRI, pheochromocytomas and paragangliomas are commonly characterized as iso or hypointense on T1-weighted images and high signal intensity on T2-weighted images, especially those with fat suppression; however, despite this finding being highly sensitive, it lacks specificity [25,26].

For lesions that are highly suspicious of PGs or PCs but express inconclusive biochemical testing results, functional imaging is recommended. Different nuclear medicine modalities can be used for functional imaging, including metaiodobenzylguanidine (MIBG) scan/scintigraphy, positron emission tomography (PET) with (18F) fluorodihydroxyphenylalanine (FDOPA), (18F) fluorodopamine (FDA), (18F) fluorodeoxyglucose (FDG), and PET with radiolabeled dodecane tetraacetic acid (DOTA) peptides [18]. MIBG scans have high specificity for detecting PCs and PGs and are helpful in detecting metastases [18]. MIGB can be labeled with 123I or 131I, with 123I being the preferred choice due to lack of beta emission, decreased radiation doses, and shorter half-life compared to 131I [22]. As a theranostic approach, 131I-MIBG provides symptomatic relief in approximately 75% of PC and PG metastatic cases [27]. FDOPA PET shows decreased specificity when compared to CT or MRI but is useful in localizing SDH-x-related PGs and PCs [18,28]. 18F-FDG PET appears to have greater sensitivity, as compared to CT, MRI, and 123/131I-MIBG, in the localization of SDHx-related PGs and PCs that are metastatic [28]. There is no agreement on the order in which radiology tests should be ordered when a PG or PC is suspected [22]. In the present case, the patient experienced symptoms of nausea, vomiting, and bloating. Subsequent CT imaging and histological analysis identified a left retroperitoneal mass characterized as a malignant paraganglioma.

As stated previously, surgical resection of the tumor is the primary treatment method for PGs [2]. Due to the potential of intraoperative catecholamine secretion and thus hemodynamic instability, pre-procedural administration of alpha-adrenergic blockades and constant monitoring of blood pressure is considered good practice [14,29]. It was chosen not to be done in this case due to physician discretion and due to the tumor being unknown to be PG. Preoperative embolization of the tumor has been shown to reduce intraoperative blood loss and reduce procedure time but comes with significant risks, including hypertensive crisis, hypotension, asystole, and even death [29-32]. The predictable hypertension that occurs when excising the tumor can be treated with various rapid agents, including sodium nitroprusside, nitroglycerin, fenoldopam, phentolamine, nicardipine, and labetalol [29]. Postoperative hypoglycemia is a common rebound effect due to the chronic excess of catecholamine production, and its effects on glucose metabolism, which the patient was experiencing before the removal of the tumor [29]. Hypertension may occur postoperatively as well [29]. Long-term follow-up is paramount for individuals diagnosed with a malignant PG, with a case of recurrence up to 22 years after tumor resection having been reported [33]. Imaging is vital during the long-term follow-up period due to the potential of an asymptomatic tumor reoccurrence [34]. In our case, the patient was successfully treated via adrenalectomy with plans to re-image in six months. Preoperative embolization and administration of alpha-adrenergic blockades were not performed because the tumor was not known to be a PG at the time of the operation.

## Conclusions

In this manuscript, we presented a case of a malignant paraganglioma in a young adult female patient. The patient presented with nausea, vomiting, and bloating and subsequent imaging showed the presence of a left retroperitoneal mass. The tumor was surgically removed, and histological analysis of the excised specimen was consistent with a paraganglioma. Due to the chance of tumor reoccurrence, the patient was started on a long-term follow-up protocol with plans to re-image in six months. Given the rarity of malignant paragangliomas, 90-95 occurrences per 400 million people, this case serves as a good reminder for physicians to always keep uncommon conditions on the differential if case findings are compatible with their etiology.
